# Proton Nuclear Magnetic Resonance Relaxation in Aqueous Sugar Solutions: Can Low-Field Nuclear Magnetic Resonance Relaxation Measurements Differentiate between “Bound” and “Free” Water?

**DOI:** 10.1021/acsphyschemau.5c00108

**Published:** 2025-11-11

**Authors:** Vasantha Gowda, Ivan Argatov, Olle Söderman, Vitaly Kocherbitov

**Affiliations:** † Department of Biomedical Science, Faculty of Health and Society, 5264 Malmö University, Malmö SE-205 06, Sweden; # Biofilms − Research Center for Biointerfaces, 5264 Malmö University, Malmö SE-205 06, Sweden; ‡ Division of Physical Chemistry, 5193 Lund University, P.O. Box 124, Lund SE-22100, Sweden

**Keywords:** proton NMR relaxation, sucrose−water system, water dynamics, solute−solvent interactions, low-field NMR

## Abstract

Understanding water interactions in complex systems is crucial, as they play a key role in fields such as biochemistry, pharmaceutical formulations, and food science. Nuclear magnetic resonance (NMR) relaxation measurements have become one of the widely used methods to visualize various water characteristics owing to their noninvasive nature and ease of use. However, unambiguous data interpretation can be challenging and potentially misleading if not carefully analyzed. One such example is the observation of multiple relaxation times, which is often linked to different water types such as “bound” and “free”. In this paper, we present a new approach for the interpretation of proton NMR relaxation data using a second-order reaction kinetics-based model. The case of first-order asymptotic analysis considering fast proton exchange is shown to be of particular relevance. The presented theory is tested using a series of sucrose–water and sucrose-D_2_O systems with varying sucrose content. The comparison of these systems reveals a biexponential behavior in both *T*_1_ and *T*_2_ relaxation times. These observations are interpreted by considering both nonexchangeable and exchangeable protons in the system, with the corresponding contribution coefficients following trends consistent with the concentrations of these proton types.

## Graphical abstract

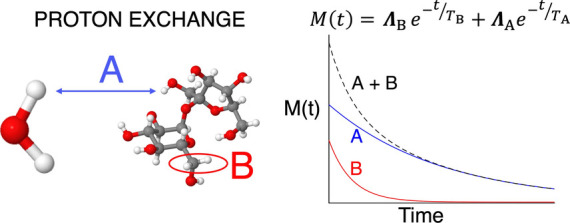

## Introduction

Unravelling the intricate dynamics of water within complex biosystems is crucial across multiple scientific domains due to profound impact of water on biomaterial properties.^1–4^ Hydration water plays a vital role in the stability, structure, dynamics, and function of proteins and other biomolecules.^5–7^ The strength of water interaction is influenced by several factors, including the chemical composition and physical properties of biomolecules, as well as various physiological conditions such as pH, temperature, pressure, and the presence of other molecules.^2,3,8^ The biomolecules’ hydrophilic or hydrophobic nature determines how strongly it attracts or repels water, while the water exchange rate—how quickly water molecules are absorbed, released, or replaced—can also impact the interaction.

Conventionally, the hydration state is presumed to be predominantly depending on the intra- and intermolecular hydrogen bond network established with the biomolecules, resulting in varying water compartments characterized by distinct strengths of hydrogen bonding. The binding of water to the biomolecules is defined by its reduced mobility as well as its anisotropic motion. The distinction between “bound” and “nonbound” has been a widely employed framework for characterizing water behaviors across various systems.^9,10^ This concept delineates distinct physical states or compartments, categorizing “bound” water into “loose” or “tight” configurations, defining unique water characteristics within these compartments.^11–13^ However, as different fields utilize varied experimental techniques to probe the biomolecular hydration process, resulting in definitions that depends on the method used. Water is generally categorized into three distinct types across various fields, based on different criteria: (i) thermal expansion, such as hydrated, interfacial, and bulk water; (ii) mobility, which includes ice-like (or tightly bound), intermediate (or loosely bound), and free water; and (iii) freezing temperature, distinguishing between nonfreezing bound water, freezing bound water, and free water.^14^ These categorizations have their own limitations, for example, thermodynamic analysis of water freezing indicates that the quantity of nonfreezing water does not necessarily correspond to the total amount of bound water.^15^ The occurrence of nonfreezing water arises from the intersection between the liquidus line and the glass transition line.^15^

The widely accepted definition of “hydration” in contemporary theory refers to solute-induced perturbations of the structure, energetics, and dynamics of the aqueous solvent.^6,16^ These perturbations are often probed using NMR parameters, with the spin–lattice relaxation time *T*_1_, transverse-relaxation time *T*_2_, and spin–lattice relaxation time in rotating frame *T*_1ρ_ (solid-state) being particularly useful for studying hydration water.^16–19^ Laage, et. al^6^ emphasized that hydration should be understood as solute-induced perturbations of water structure, energetics, and dynamics with graded, heterogeneous slowdowns, not as a binary “bound” vs “free” state. This view is consistent with earlier NMR studies on sugar solutions^20–23^ and more recent work^24^ showing that hydration dynamics are strongly environment-dependent. Recent NMR studies on trehalose and glucose solutions^25^ and in porous silica gel^26^ further confirm that water relaxation reflects a continuum of exchange rates and local environments, not two distinct populations. However, in much of the literature, relaxation (*T*_1_/*T*_2_) measurements of hydrated biomolecules yield a multicomponent relaxation profile, which are often misinterpreted as evidence for distinct water “bound” and “unbound” or “free” water states.^11,27,28^ However, can NMR relaxation measurements effectively differentiate between “bound” and “unbound” water?

In a static magnetic field *B*_0_, nuclear spin levels (2*I* + 1) adopt a Boltzmann distribution with a slight population excess in the lower-energy state. During an NMR experiment, the equilibrium state is perturbed by the application of a second oscillating radiofrequency field (*B*_1_), driving transitions and energy absorptions.

Relaxation of nuclear spins back to equilibrium occurs via four primary mechanisms:^29^ (i) dipolar coupling to thermally driven local field fluctuations (lattice motion), (ii) molecular reorientations and translation, (iii) proton exchange, and (iv) interactions with paramagnetic centers. Energy transfer from spins to the lattice defines the longitudinal (spin–lattice) relaxation time (*T*_1_); after the applied RF field along *B*_1_ is switched-off, *M*_*z*_ recovers to equilibrium with time constant *T*_1_, while *M*_*xy*_ decays with *T*_2_.

*T*_1_ and *T*_2_ probe dynamics at different spectral regions: generally, high-frequency motions contribute to both, whereas low frequency processes influence mainly the *T*_2_ relaxation, causing *T*_2_ ≪*T*_1_ when the condition ωτ_c_ ≪1 where ω is the Larmor frequency and τ_c_ is the correlation time. In liquids, rapid molecular motions average dipolar fields, reducing both ${T_{1}}^{-1}$ and ${T_{2}}^{-1}$ . Chemical exchange will affect both *T*_1_ and *T*_2_. *T*_1_ is affected because energy is transferred from one nucleus to another. For example, if there are more nuclei in the excited state of water (A*), and a normal Boltzmann distribution in sucrose (*B*), exchange will force the excess energy from A* into *B*. *T*_2_ is affected because phase coherence of the transverse magnetization is not preserved during chemical exchange.

In the hydration layer of macromolecules, magnetic interactions between water protons are only partially averaged, with the extent determined by specific water-macromolecule interactions. These include proton exchange, reorientational dynamics, and translational diffusion of water molecules through regions of varying structural order. In contrast, free or bulk water exhibits rapid isotropic motion that effectively averages out dipolar couplings, dominating the relaxation behavior. It is often associated with the notion that water molecules interacting with solutes, like sugars or other molecules, experience different environments, leading to distinct NMR relaxation times. However, this conceptual framework has limitations for its oversimplification, with debates on its ability to accurately capture the intricate dynamics of water within heterogeneous systems.^6^

We put forward an alternative perspective that the observed differences in water characteristics, e.g., in ^1^H *T*_1_ and *T*_2_ relaxation times, are primarily attributed to the varying rates of chemical exchange between water molecules and solutes, such as sugars, rather than the existence of distinct compartments of “bound” and “nonbound” water. The rate at which these exchanges occur can alone dictate the observed variations in the relaxation times. For instance, the water molecules are actively engaged in rapid intra molecular exchange processes as well as (not so rapid) with the exchangeable protons of the solute molecules. Additionally, there’s a third scenario wherein nonexchangeable hydrogen within the sugar also undergo relaxation, however, that will not impact the water relaxation significantly. The first two types of dynamic exchange process, intramolecular and water-solute exchange, can cause the protons within the water molecules to experience differing environments, resulting in the diverse relaxation times observed experimentally.

The aim of this work is to demonstrate the direct generalization of second-order reaction kinetic equations to incorporate the effect of chemical exchange and to illustrate the biexponential decay of the longitudinal and transverse magnetizations without considering any distinction between “bound” or “non-bound” water. Our model directly addresses these issues by demonstrating, through explicit exchange-based analysis in H_2_O, D_2_O, and partially deuterated sucrose samples, that the observed relaxation can be explained without invoking a separate bound-water fraction, thereby providing a more accurate framework for future research.

## Materials and Methods

### Experimental Details

Crystalline sucrose was purchased from Sigma-Aldrich and used as obtained. Sucrose–water systems with a fixed water content (1 g), and increasing sucrose composition from 0 to 70 wt % in Milli-Q water, were analyzed using a Spin Track TD-NMR analyzer operating at 18.27 MHz. It was ensured that no solid sucrose remained in the 70 wt % solution. The solution pH ranged between 6.5 and 7.5 (approximately neutral) across the different concentrations.

To decrease concentration of exchangeable protons, crystalline sucrose was dissolved in D_2_O (20 wt % sucrose - 80 wt % D_2_O solution) and then freeze-dried. The obtained partially deuterated sucrose was dissolved in D_2_O. Apart from nonexchangeable protons, the samples obtained this way (denoted as FD-D_2_O) still contained a small number of exchangeable protons (see Table S3). The samples were placed in a 10 mm diameter NMR tube. Measurements were conducted at 25 °C, allowing the samples to equilibrate for 15 min. Further extension of the thermostatting time did not impact the measurement outcomes. Proton spin–lattice relaxation times (*T*_1_) were measured using the saturation recovery method by adding 32-time intervals ranging from 4 ms to 30 s, a relaxation delay of 2.5 s, and 4 scans. The spin–spin relaxation times (*T*_2_) were determined using the Carr–Purcell–Meiboom–Gill (CPMG) pulse sequence with specific parameters: a 3.3 μs duration for the 90° pulse, a 300 μs-3000 μs time interval (τ) between the 90° and 180° pulses, and a range of 3000 echoes on the decay curve. Each sample underwent three identical measurements, accumulating 32 scans with a 15 s delay between scans.

The ^1^H *T*_1_ and *T*_2_ relaxation data were analyzed using a nonlinear least-squares fitting procedure implemented in MATLAB (MathWorks Inc.). The relaxation curves were fit to biexponential models of the forms:

$$\frac{M_{z}(t)}{M_{Z}^{\infty }}=1-a_{1s}e^{-t/T_{1s}}-a_{1f}e^{-t/T_{1f}}$$ 1

$$\frac{M_{xy}(t)}{M_{xy}^{0}}=a_{2s}e^{-t/T_{2s}}+a_{2f}e^{-t/T_{2f}}$$ 2

where *T*_1s_ and *T*_1f_ are the two *T*_1_ relaxation times, while *T*_2s_ and *T*_2f_ correspond to the two *T*_2_ relaxation times. The respective populations (normalized) of the slow-relaxing and fast-relaxing components are denoted *a*_1s_ and *a*_1f_, respectively. An offset constant, *y*_0_, is included in the MATLAB fitting to account for residual signal contributions arising from imperfect pulse calibration and baseline drifts. Initial parameter estimates were chosen based on signal amplitude and time scale and bounded within physically meaningful limits. The fitting was performed using the lsqcurvefit function in MATLAB, with convergence criteria set by increasing the maximum number of iterations and function evaluations. Goodness-of-fit was assessed using the coefficient of determination (*R* ^2^) and the root-mean-square error (RMSE). Each *T*_1_ and *T*_2_ measurement was performed in triplicate to account for experimental variability. The reported values are means of the triplicate measurements, with errors representing the standard deviation across replicates. Each data set was fit independently and we did not combine individual fitting errors with replicate variability. Representative saturation recovery (*T*_1_) relaxation curves for the 50 wt % sucrose solution, along with 80% confidence intervals (CIs), were fitted and plotted for each of the three independent trials. These sample plots are provided in the Supporting Information (Figure S2) to illustrate the fitting quality and variability across replicates.

## Theory

### Second-Order Kinetics Model of Proton Exchange

In nonionic systems such as water-carbohydrate, to preserve electrical neutrality of the molecules, a transfer of a proton from molecule *A* to *B* implies a transfer of another proton from molecule *B* to *A*. This interpretation aligns with the cyclic concerted mechanism proposed by Harvey and Symons^30^ and Hills,^31^ where proton exchange occurs via coordinated hydrogen-bond jumps that enable rapid proton switching between water and sugar hydroxyl groups without charge separation. Hence the chemical exchange of hydrogen atoms between two electrically neutral molecules in a solution follows second order kinetics:

$$A+B^{*}\overset{k_{\mathrm{ex}}}{\to }A^{*}+B$$ 3

$$A^{*}+B\overset{k_{\mathrm{ex}}}{\to }A+B^{*}$$ 4

Here “*A*” and “*B*” represents the exchangeable ground states (β) protons in the two molecules, while “A*” and “B*” denote the excited state (α) protons. Without losing generality, below we will consider “*A*” and “A*” as protons in water molecules while “*B*” and “B*” as protons in sucrose molecules. In an exchange process, “B*” hydrogen of sucrose exchanges with “*A*” hydrogen of water and the “A*” hydrogen on water exchanges with sucrose in the forward and backward reactions. Knowing that the influence of the proton state on the chemical properties of molecules is minor, we assume that the *k*_ex_ values of the forward and backward reactions are equal.

The rate of chemical exchange corresponding to eqs 3 and 4 can be described using a set of two first-order differential equations:

$$\frac{dA^{*}}{dt}=k_{\mathrm{ex}}\cdot A\cdot B^{*}-k_{\mathrm{ex}}\cdot A^{*}\cdot B$$ 5

$$\frac{dB^{*}}{dt}=k_{\mathrm{ex}}\cdot A^{*}\cdot B-k_{\mathrm{ex}}\cdot A\cdot B^{*}$$ 6

The total concentrations of protons from water (*A* ^tot^) and sucrose (*B* ^tot^), can be expressed as follows:

$$A^{\mathrm{tot}}=A^{*}+A\;\;\;\;B^{\mathrm{tot}}=B^{*}+B$$ 7

Substituting eq 7 for “*A*” and “*B*” in eqs 5 and 6, respectively:

$$\frac{dA^{*}}{dt}=k_{\mathrm{ex}}(A^{\mathrm{tot}}\cdot B^{*}-B^{\mathrm{tot}}\cdot A^{*})$$ 8

$$\frac{dB^{*}}{dt}=k_{\mathrm{ex}}(B^{\mathrm{tot}}\cdot A^{*}-A^{\mathrm{tot}}\cdot B^{*})$$ 9

### Longitudinal Magnetization

The net proton longitudinal magnetizations due to sugar (*M*_*z*,*B*_) and water (*M*_*z*,*A*_) are proportional to the population difference between their respective spin states:

$$M_{z,B}=\left(B-B^{*}\right)\mu \;\mathrm{and}\;M_{z,A}=\left(A-A^{*}\right)\mu$$ 10

where μ is the proton magnetic moment.

Substituting eq 7, one obtains expressions for concentrations of protons in excited states:

$$A^{*}=\frac{A^{\mathrm{tot}}}{2}-\frac{M_{z,A}}{2\mu }\;\mathrm{and}\;B^{*}=\frac{B^{\mathrm{tot}}}{2}-\frac{M_{z,B}}{2\mu }$$ 11

Therefore, eqs 8 and 9 can be expressed in terms of magnetization as follows:

$$\frac{dM_{z,A}}{dt}=k_{B}\cdot M_{z,B}-k_{A}\cdot M_{z,A}$$ 12

$$\frac{dM_{z,B}}{dt}=-k_{B}\cdot M_{z,B}+k_{A}\cdot M_{z,A}$$ 13

where *k*_*A*_= *k*_ex_ · *B* ^tot^ and *k*_*B*_= *k*_ex_ · *A* ^tot^, given that *A* ^tot^ and *B* ^tot^ are constants in an NMR experiment.

Adding the longitudinal relaxation term in the absence of chemical exchange (*R*_1_ *=* 1/*T*_1_), to eqs 12 and 13,

$$\frac{dM_{z,A}}{dt}=R_{1,A}(M_{z,A}^{\infty }-M_{z,A})-k_{A}\cdot M_{z,A}+k_{B}\cdot M_{z,B}$$ 14

$$\frac{dM_{z,B}}{dt}=R_{1,B}(M_{z,B}^{\infty }-M_{z,B})-k_{B}\cdot M_{z,B}+k_{A}\cdot M_{z,A}$$ 15

where *M*_*z*,*B*_^∞^ and *M*_*z*,*A*_^∞^ represent the equilibrium longitudinal magnetizations due to sugar and water protons, respectively. Equations 14 and 15 constitute a system of differential equations that describe longitudinal relaxation when the proton exchange obeys eqs 3 and 4. This system of equations formally coincides with the conventional Bloch–McConnell equations^32–34^ but the rate coefficients *k*_*A*_ and *k*_*B*_ are here defined as concentration-dependent quantities (*k*_*A*_= *k*_ex_ · *B* ^tot^, *k*_*B*_= *k*_ex_ · *A* ^tot^), explicitly reflecting the second-order nature of the underlying proton-exchange process. Unlike the Bloch–McConnell formulations, which assumes first-order rate constants, our approach directly links the observed relaxation behavior to the actual molecular concentrations of the exchanging species, providing a more physically realistic description of water-sucrose system. Nonetheless, due to a similarity of the mathematical structure, the solution of the differential equation has a similar form and is given below:

$$M_{z,A}\left(t\right)=-\Lambda_{1}e^{-\lambda_{1}t}-\Lambda_{2}e^{-\lambda_{2}t}+M_{z,A}^{\infty }$$ 16

$$M_{z,B}(t)=-\Lambda_{1}\frac{\left(r_{A}-\lambda_{1}\right)}{k_{B}}e^{-\lambda_{1}t}-\Lambda_{2}\frac{\left(r_{A}-\lambda_{2}\right)}{k_{B}}e^{-\lambda_{2}t}+M_{z,B}^{\infty }$$ 17

where the normalized longitudinal magnetization recovery for the two exchanging pools can be expressed as

$$\frac{M_{z,A}(t)+M_{z,B}(t)}{M_{z,A}^{\infty }+M_{z,B}^{\infty }}=1-\frac{\Lambda_{1}(k_{A}+r_{A}-\lambda_{1})}{k_{B}(M_{z,A}^{\infty }+M_{z,B}^{\infty })}e^{-\lambda_{1}t}-\frac{\Lambda_{2}(k_{A}+r_{A}-\lambda_{2})}{k_{B}(M_{z,A}^{\infty }+M_{z,B}^{\infty })}e^{-\lambda_{2}t}$$ 18

and

$$\lambda_{1}=\frac{1}{2}\{(r_{A}+r_{B})-\sqrt{(r_{A}-r_{B}{)}^{2}+4k_{A}k_{B}}\}$$ 19

$$\lambda_{2}=\frac{1}{2}\{(r_{A}+r_{B})+\sqrt{(r_{A}-r_{B}{)}^{2}+4k_{A}k_{B}}\}$$ 20

$$\Lambda_{1}=-\frac{M_{z,A}^{\infty }}{\left(\lambda_{2}-\lambda_{1}\right)}(R_{1,A}-\lambda_{2})$$ 21

$$\Lambda_{2}=-\frac{M_{z,A}^{\infty }}{\left(\lambda_{2}-\lambda_{1}\right)}(\lambda_{1}-R_{1,A})$$ 22

$$r_{A}=k_{A}+R_{1,A}\;\mathrm{and}\;r_{B}=k_{B}+R_{1,B}$$ 23

Observe that, while the second-order kinetics eqs 3 and 4 are symmetric, formulas [Disp-formula eq16] and 17-derived using a standard Gaussian elimination scheme-are not. In particular, the expression for *M*_*z*,*B*_(*t*) contains the exchange coefficient *k*_*B*_ in the denominator (see eq 17). The efficiency of the symmetrical form of the solution was emphasized^35^ in solving the inverse problem for the Bloch–McConnell equations.

### Transverse Magnetization

In comparison to eqs 12 and 13, the kinetic equations in terms of transverse magnetization can be expressed as follows:

$$\frac{dM_{xy,A}}{dt}=k_{B}\cdot M_{xy,B}-k_{A}\cdot M_{xy,A}$$ 24

$$\frac{dM_{xy,B}}{dt}=-k_{B}\cdot M_{xy,B}+k_{A}\cdot M_{xy,A}$$ 25

By adding the *T*_2_ relaxation in the absence of exchange:

$$\frac{dM_{xy,A}}{dt}=-(R_{2,A}-i\omega_{A})M_{xy,A}-k_{A}\cdot M_{xy,A}+k_{B}\cdot M_{xy,B}$$ 26

$$\frac{dM_{xy,B}}{dt}=-(R_{2,B}-i\omega_{B})M_{xy,B}-k_{B}\cdot M_{xy,B}+k_{A}\cdot M_{xy,A}$$ 27

in the absence of the radio frequency field. Here $\frac{dM_{xy,j}}{dt}$ is the time derivative of the transverse magnetization *M*_*xy*,*j*_ in site *j*, where *j* = *A*, *B*. In this context *R*_2,*j*_ and ω_*j*_ refer to the corresponding transverse relaxation rate of the macroscopic magnetic moment, and the Larmor frequency, respectively, in the absence of exchange.

The general solutions of eqs 26 and 27 are obtained by applying the following substitutions *M*_*z*,*B*_^∞^ → 0 and *R*_1,*j*_ → *R*_2,*j*_ – *i*ω_*j*_ to eqs 16 and 17 as follows:

$$M_{xy,A}\left(t\right)=\Theta_{1}e^{-\vartheta_{1}t}+\Theta_{2}e^{-\vartheta_{2}t}$$ 28

$$M_{xy,B}\left(t\right)=-\frac{\Theta_{1}\left(\vartheta_{1}-r_{2A}\right)}{k_{B}}e^{-\vartheta_{1}t}-\frac{\Theta_{2}\left(\vartheta_{2}-r_{2A}\right)}{k_{B}}e^{-\vartheta_{2}t}$$ 29

where,

$$\vartheta_{1}=\frac{1}{2}\{(r_{2A}+r_{2B})-\sqrt{(r_{2A}-r_{2B}{)}^{2}+4k_{A}k_{B}}\}$$ 30

$$\vartheta_{2}=\frac{1}{2}\{(r_{2A}+r_{2B})+\sqrt{(r_{2A}-r_{2B}{)}^{2}+4k_{A}k_{B}}\}$$ 31

$$r_{2A}=R_{2,A}-i\omega_{A}+k_{A}$$ 32

$$r_{2B}=R_{2,B}-i\omega_{B}+k_{B}$$ 33

$$\Theta_{1}=-\frac{M_{xy,A}^{0}(\vartheta_{2}-r_{2A})+k_{B}M_{xy,B}^{0}}{\left(\vartheta_{1}-\vartheta_{2}\right)}$$ 34

$$\Theta_{2}=M_{xy,A}^{0}+\frac{M_{xy,A}^{0}(\vartheta_{2}-r_{2A})+k_{B}M_{xy,B}^{0}}{\left(\vartheta_{1}-\vartheta_{2}\right)}$$ 35

The equations derived above indicate that the magnetizations in both *T*_1_ and *T*_2_ relaxations should exhibit biexponential decay (eqs 16, 17, 28, and 29) without invoking any assumption of free and bound water. In the case of very fast proton exchange, the situation may differ and will be addressed in the Discussion section. To validate the equations derived here, the following sections present and analyze proton NMR relaxation data for a simple liquid system-sucrose in water. To investigate the effect of proton concentration, a partially deuterated system (sucrose in D_2_O) is also considered.

## Results

### Longitudinal (*T*_1_) Relaxation in the Water–Sucrose System

The longitudinal (*T*_1_) relaxation times of protons in sucrose-water solutions were measured in the concentration range of 0–70 wt % of sucrose using the saturation recovery pulse sequence (Figure S1a).

Although in certain studies, (e.g.,^28^), proton relaxation in sucrose-water system is considered as a single exponential process. However, our experimental results indicate proton relaxation in these systems is more accurately described using biexponential approximations, as demonstrated by the residual plot for the 50 wt % sucrose-water solution shown in Figure 1a. Hence, the magnetization was fitted by a biexponential function, see eq 1, except for sucrose concentrations below 10 wt %, where the data fit well to a single exponential function. Both the fast (*T*_1f_) and slow (*T*_1s_) relaxation times gradually decreased with increasing sucrose concentration (see Figure 2a, blue lines). The relative contribution of the fast-relaxing component (*a*_1f_) increased with sucrose content, while that of the slow relaxing component (*a*_1s_) decreased proportionally (see Figure 2b).

**Figure 1:**
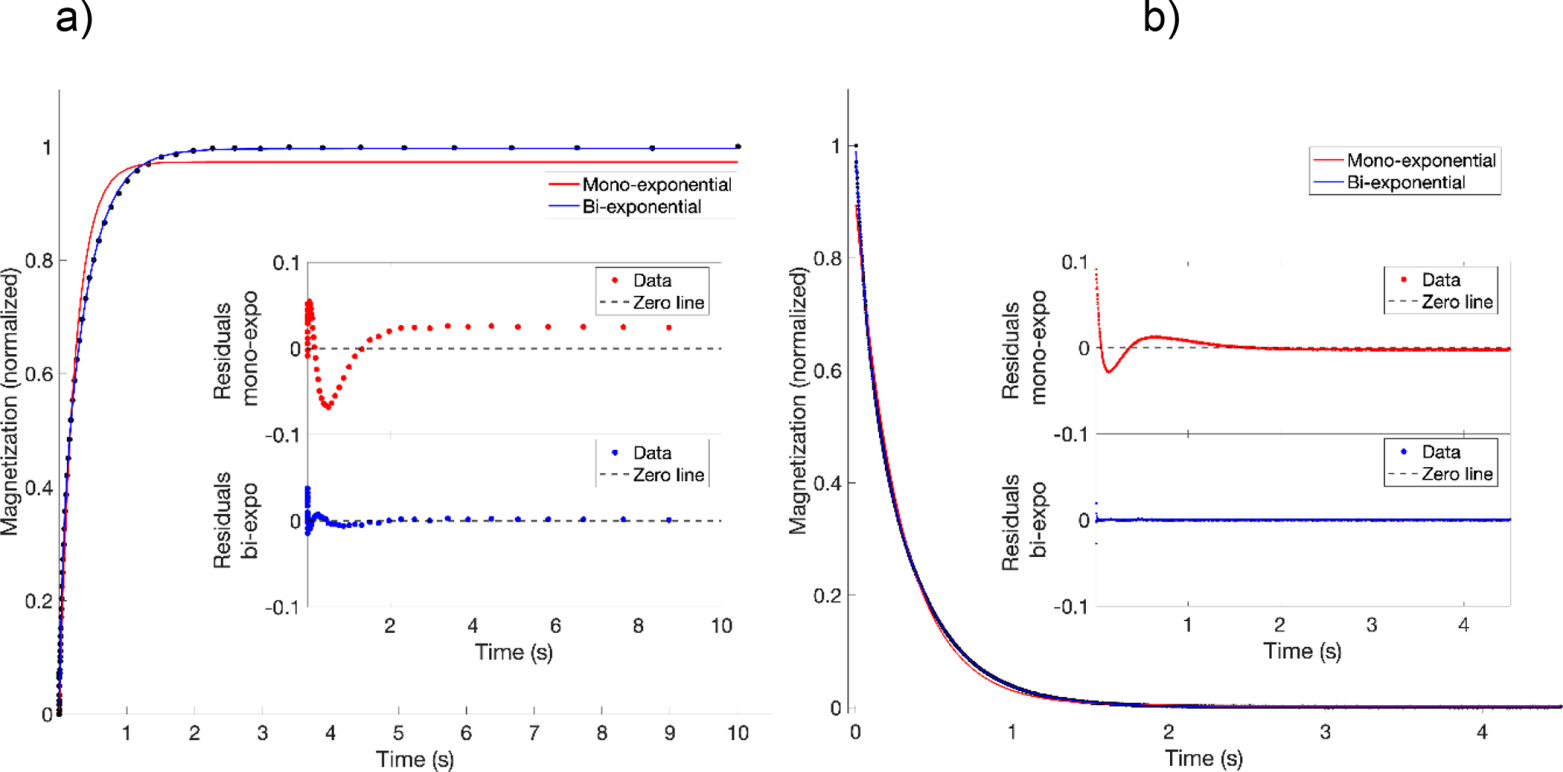
(a) Saturation recovery ^1^H *T*_1_ and (b) CPMG *T*_2_ relaxation measurements for a 50 wt % water-sucrose solution at 25 °C, showing both single- and double-exponential least-squares fits. Insets show the residuals for both fitting models.

**Figure 2:**
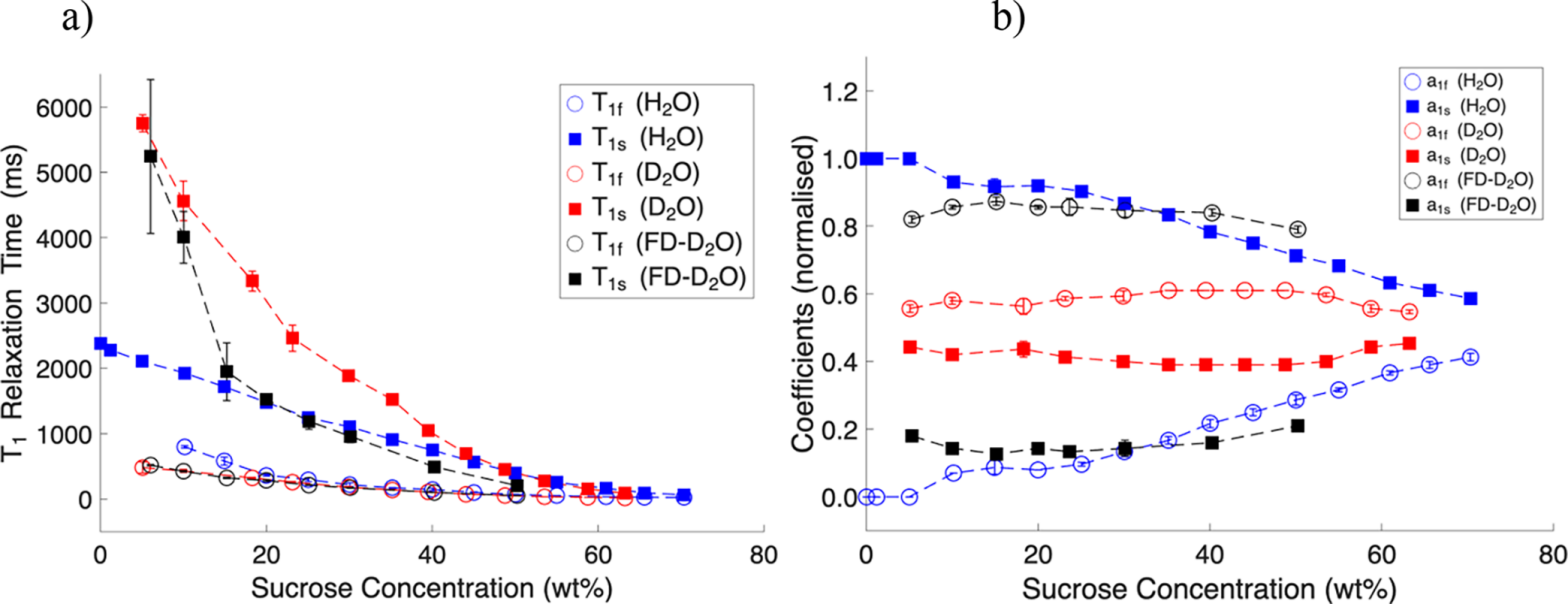
Two-component ^1^H *T*_1_ relaxation times (ms) (a) and coefficients (b) in sucrose-water systems as a function of sucrose concentration (wt %).

In D_2_O-sucrose solutions, *T*_1_ relaxation times of protons displayed biexponential behavior across the concertation range of 5–65 wt %. Notably, the *T*_1s_ values observed in D_2_O (Figure 2a, red squares) were significantly higher than those observed in Milli-Q water solutions for sucrose concentrations below 40 wt %, while the *T*_1f_ values remained comparable for both systems across the studied concentration range (see also Table S1 in the Supporting Information). Interestingly, in contrast with the normal water case, the relaxation component contributions (*a*_1f_ ≈ 0.6 and *a*_1s_ ≈ 0.4) were almost independent of concentration (see Figure 2b, red symbols).

The *T*_1_ relaxation times of protons in FD-D_2_O samples are shown in Figure 2a (black symbols). Measurements were conducted for concentrations ranging from 5 to 30 wt % freeze-dried sucrose (see Table S1 in the Supporting Information). The *T*_1f_ values followed a similar trend to D_2_O-sucrose solutions, the *T*_1s_ values aligned more closely with trends in Milli-Q water-based sucrose solutions. Similarly to sugar dissolved in D_2_O, in FD-D_2_O samples, the coefficients remained constant, with a significantly higher fast-relaxing contribution (*a*_1f_ ≈ 0.85) compared to the slow-relaxing contribution (*a*_1s_ ≈ 0.15) (see Figure 2a,b).

### Transverse (*T*_2_) Relaxation in the Water–Sucrose System

The transverse relaxation was studied using the CPMG pulse sequence across sucrose the concentration range of 0–70 wt % (see Figure S1b). Consistent with the behavior of *T*_1_ relaxation, the CPMG *T*_2_ relaxation curves is more accurately captured by biexponential fitting, as illustrated by the residual plot for the 50 wt % sucrose-water solution in Figure 1b. The transverse (*T*_2_) relaxation times of protons in sucrose-water solutions were calculated using the biexponential equation eq 2, where contributions are classified as fast *T*_2f_ and slow *T*_2s_ relaxations and presented in Figure 3 and Table S2 in the Supporting Information. In general, the *T*_2_ relaxation times follow a similar trend as *T*_1_ relaxation times, decreasing with the sucrose concentration. The relative contribution coefficients *a*_2f_ and *a*_2s_ also quantitatively follow the coefficients of longitudinal relaxation.

**Figure 3:**
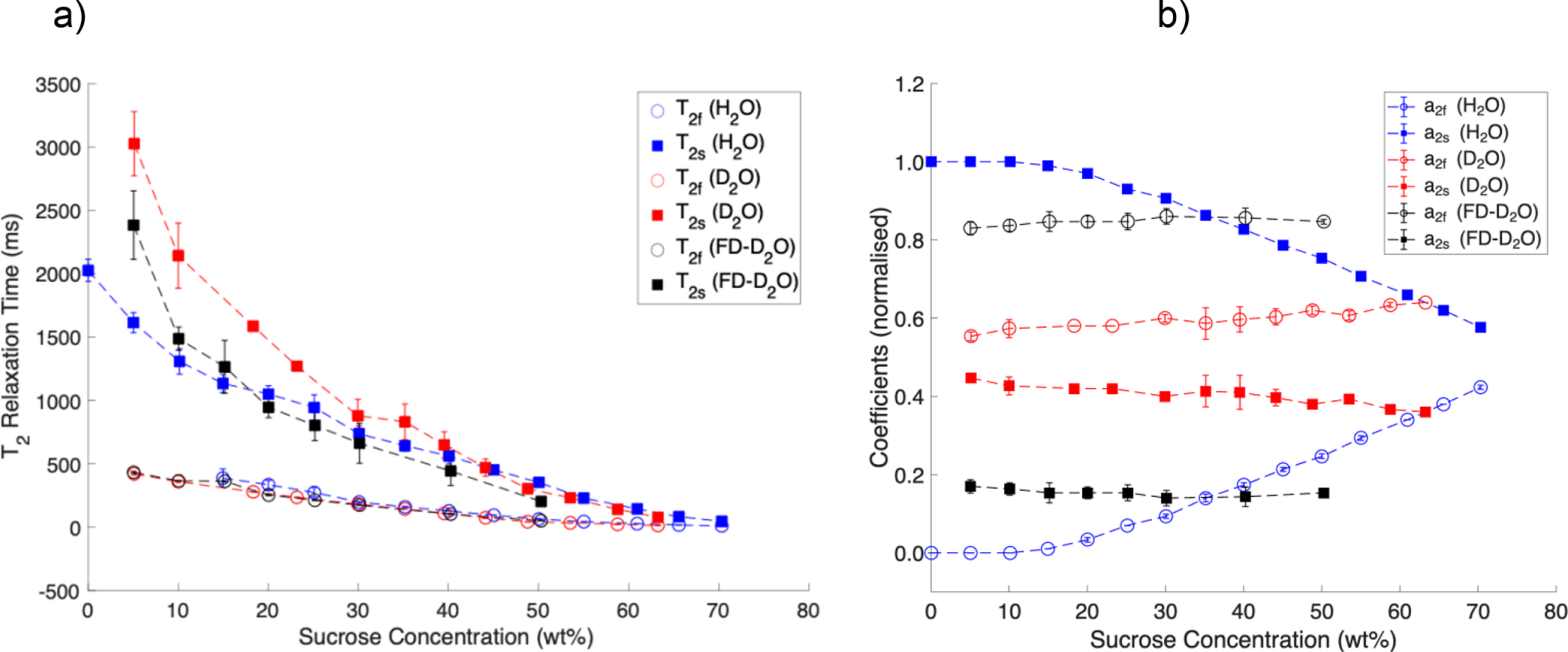
Two-component ^1^H CPMG *T*_2_ relaxation times (ms) (a) and their populations (b) in sucrose-water (Milli-Q) system as a function of sucrose concentration (wt %).

## Discussion

NMR studies of the interactions between water and carbohydrates, as well as other biomolecules, are abundant. Their number further increased with the rapid adoption of time-domain NMR techniques, which enable simple and cost-effective analysis of proton relaxation. In many of these studies, water binding is evaluated by attributing the slow and fast relaxation components (as illustrated by eqs 1 and 2) to “free” and “bound” water, respectively. Although it is often noted that proton exchange can influence the results, a consistent theory that correctly describes NMR relaxation in homogeneous aqueous systems with proton exchange-while explicitly accounting for the bimolecular nature of the exchange-has not yet been presented in the literature. In this section, we analyze the experimental data presented in the Results section using the equations derived in the Theory section.

As mentioned above, proton exchange results in biexponential relaxation behavior even in the absence of any difference between free and bound water. However, this does not necessarily mean that the relaxation time data presented in Figures 2 and 4 correspond to λ_1,2_ (eqs 19 and 20) and ϑ_1,2_ (eqs 30 and 31), respectively. It is important to note that a sucrose molecule contains 14 nonexchangeable protons and 8 hydroxyl protons that undergo exchange with the solvent (water). In the analysis presented in the Theory section, we explicitly considered contributions only from exchangeable protons (generally producing biexponential behavior), while contributions from nonexchangeable protons-which can introduce a third exponential decay component-should also exist.^31^ To figure out the nature of the biexponential behavior observed in the experiments, we calculated fractions of exchangeable (*x*_p_^exch^) and nonexchangeable (*x*_p_^non^) protons under the three experimental conditions used in this study (H_2_O, D_2_O and FD-D_2_O). These calculations were based on component concentrations using formulas S20–S25 (Supporting Information), and the results are plotted as a function of sucrose wt % (see Figure 4).

**Figure 4:**
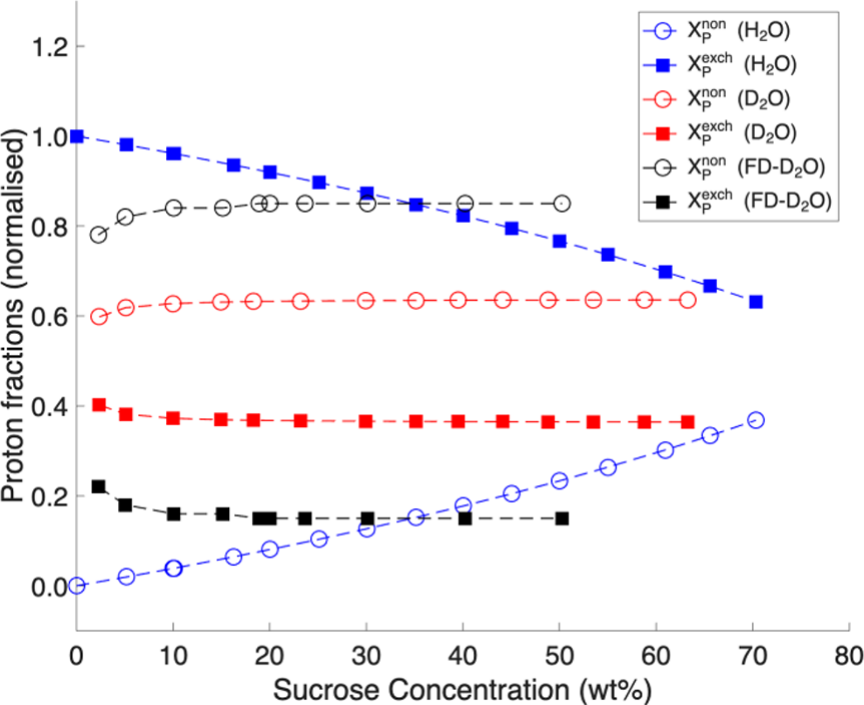
Predicted fractions of exchangeable and nonexchangeable protons in sucrose-H_2_O, sucrose-D_2_O and FD sucrose-D_2_O systems, calculated using eqs S20–S27.

The calculated exchangeable and nonexchangeable proton concentrations for sucrose-H_2_O and sucrose-D_2_O systems across a range of sucrose contents are shown in Figure 4 (also see Table S3 in the Supporting Information). Clearly, calculated proton fractions exhibit the same trends as in experiments and the values closely align with the experimental data presented in Figures 2b and 3b. In particular, in H_2_O, the two relative contributions of fast and slow signals exhibit opposite behaviors: the fraction of the fast-relaxing component increases with sucrose content, whereas the fraction of the slow-relaxing component decreases. Likewise, concentration of nonexchangeable protons increases with sucrose content (wt %), while the total concentration of exchangeable protons decreases.

In contrast, for systems prepared in D_2_O, the contributions of relaxation components remain nearly constant across all concentrations (Figures 2b and 3b). Similarly, in the D_2_O system, both nonexchangeable and exchangeable proton concentrations remain relatively constant (Figure 4).

Hence analysis of ratios *a*_1f_/*a*_1s_ and *a*_2f_/*a*_2s_ in three systems suggest that the fast-relaxing component primarily originates from nonexchanging protons. This conclusion is supported by several observations:

- The relative contribution from the fast components clearly grows upon the increase of D_2_O content in the sequence H_2_O → D_2_O → FD-D_2_O. In H_2_O, the fast-relaxing component is consistently smaller than the slow-relaxing component, whereas in D_2_O, the fast-relaxing component becoming dominant.
- The relative contributions of the fast components are nearly independent of water content in both D_2_O and FD-D_2_O samples, since the addition of D_2_O does not affect the amounts of exchangeable protons.
- The observed contributions closely align with calculated proton concentrations, both in trend and magnitude.

Hence, the other, slow-relaxing component originates from exchangeable protons. The theory presented above, however, predicts the existence of two contributions in both the *T*_1_ and *T*_2_ experiments (eqs 21 and 22 and eqs 34 and 35, respectively). The absence of the second, “exchangeable”, component can be explained by the asymptotic analysis for the case of fast proton exchange.

Assuming that *R*_1,*A*_ and *R*_1,*B*_ ≪ *k*_*A*_ and *k*_*B*_, where *k*_*A*_= *k*_ex_ *B* ^tot^ and *k*_*B*_= *k*_ex_ *A* ^tot^ one can show (see the Supporting Information for details) that

$$\lambda_{1}≅x_{A}R_{1,A}+x_{B}R_{1,B}$$ 36

$$\lambda_{2}≅k_{\mathrm{ex}}(A^{\mathrm{tot}}+B^{\mathrm{tot}})+x_{B}R_{1,A}+x_{A}R_{1,B}$$ 37

where *x*_*A*_ = *A* ^tot^/(*A* ^tot^ + *B* ^tot^) is the fraction of water protons and analogously *x*_*B*_ is the fraction of sugar protons in the total pool of exchangeable protons (see Tables S4–S6 in the Supporting Information). Due to inequality listed above, λ_1_ < λ_2_, and moreover, disregarding the last two terms in eq 37, λ_2_ ≅ *k*_ex_(*A* ^tot^ + *B* ^tot^). In other words, λ_2_ depends on the chemical exchange rate rather than on NMR relaxation. On the other hand, for this asymptotic case one can show (see the Supporting Information) that

$$a_{1s}≅1,a_{1f}≅0$$ 38

$$a_{2s}≅1,a_{2f}≅0$$ 39

This shows that in the limit of fast exchange, the contributions associated with fast components (λ_2_ and ϑ_2_ for *T*_1_ and *T*_2_ relaxations, respectively) vanish. In the asymptotic case of fast proton exchange (large *k*_*A*_ and *k*_*B*_), our analysis shows that the system relaxes with a single effective relaxation time. This is given by the weighted mean of the component relaxation rates, in agreement with the prediction of the Bloch–McConnell equations (eq 32) It is worth noting that despite linear dependence in eq 36, *R*_1,*A*_ and *R*_1,*B*_ are expected to be dependent on water content and hence λ_1_ dependence on water content should not necessarily be linear.

In Figure 5, we compare the experimental relaxation times *T*_1_ and *T*_2_ with theoretical values derived from both exact solutions (eqs 19 and 20) and the fast exchange approximation (eqs 36 and 37). The total exchangeable proton concentrations (mol/m^3^) from solvent and sucrose were calculated explicitly using expressions S24–S29 (Supporting Information). For sucrose in H_2_O, both models yield nearly identical predictions for 1/λ_1_ while 1/λ_2_ approaches zero. This result reflects the dominant influence of the large water proton reservoir and the relatively minor contribution of sucrose hydroxyl protons. These findings demonstrates that when the proton pool is abundant, the fast-exchange approximation reliably describes the proton relaxation behavior.

**Figure 5:**
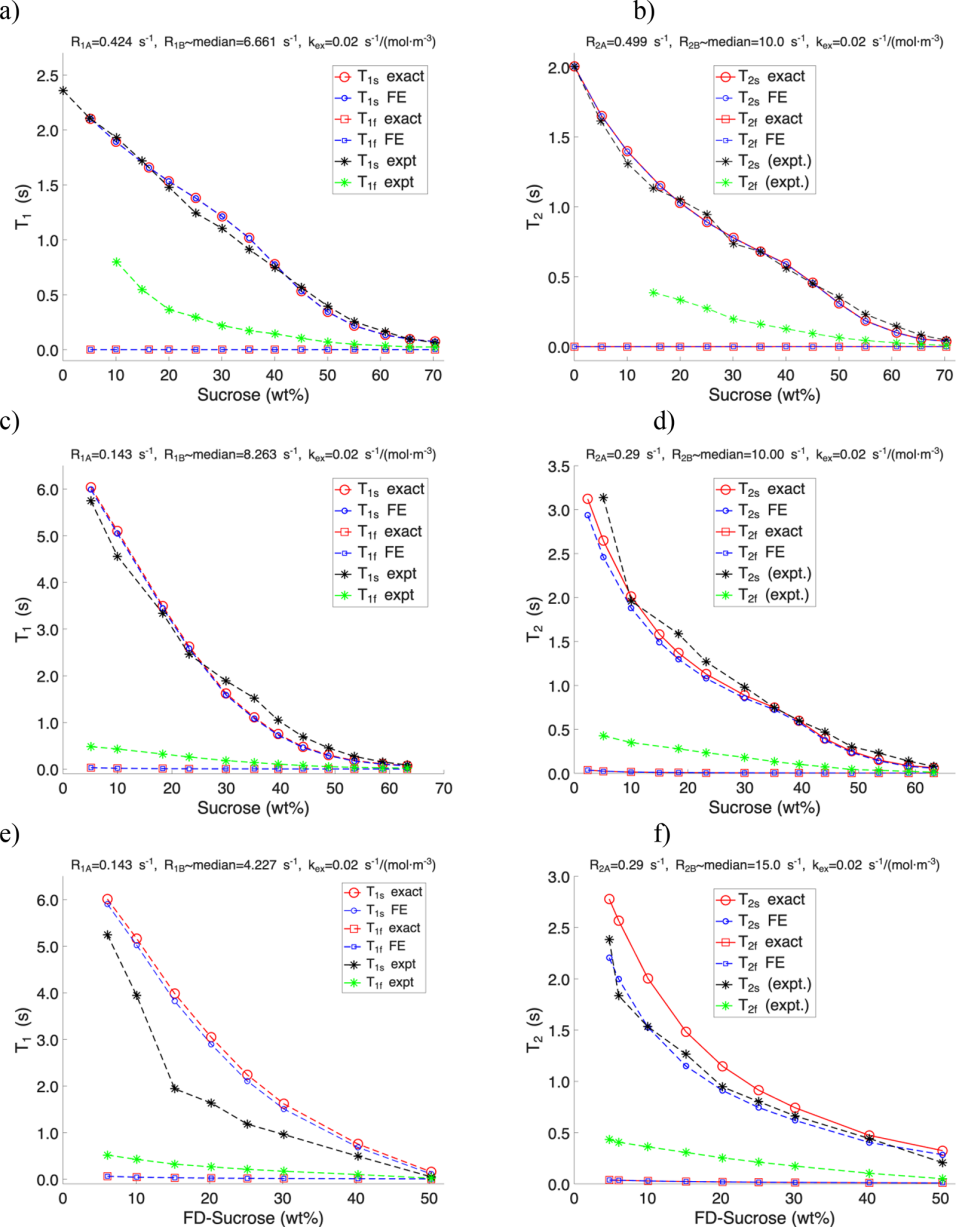
Comparison of experimental relaxation times *T*_1_ and *T*_2_ with theoretical values derived from 1/λ_1_ and 1/λ_2_ exact solutions and the fast exchange approximation. Panels (a, b) correspond to *T*_1_ and *T*_2_, respectively, for sucrose in H_2_O; panels (c, d) for sucrose in D_2_O; and panels (e, f) for freeze-dried sucrose reconstituted in D_2_O.

In contrast, for sucrose in D_2_O, where the solvent proton concentration is minimal, small but consistent deviations appear between the exact and fast-exchange approximate model. In this case, sucrose hydroxyl protons contribute significantly to the exchangeable pool, leading to greater discrepancies between theory and experiment, especially for *T*_2_.

For the freeze-dried sucrose samples reconstituted in D_2_O, partial deuteration of hydroxyl groups further reduces the concentration of exchangeable protons. The experimental data for these samples show clear deviations from both exact and approximate predictions, reflecting changes in the balance between solvent- and solute-derived exchangeable sites. These results demonstrate the sensitivity of relaxation behavior to solvent isotope composition and sample preparation and highlight the limitations of the fast-exchange approximation under proton-depleted conditions.

The correlation between 1/λ and the exchange rate constant *k*_ex_ (s^–1^·mol^–1^ ·m^3^), as well as between 1/λ and the mole fraction of sucrose (*x*_*B*_), further illustrates the distinct effects of solvent environment and sample preparation on exchange dynamics (Figure 6). For sucrose in H_2_O*,* 1/λ_1_is highly sensitive to sucrose concentration, particularly in the intermediate exchange regime 10^–4^–10^–2^ s^–1^ per mol/m^3^, whereas in the fast exchange limit it remains nearly constant. In contrast, 1/λ_2_ approaches zero across this range, indicating that relaxation is almost entirely governed by the dominant water proton reservoir. At very low exchange rates (*k*_ex_ < 10^–4^ s^–1^·mol^–1^·m^3^,) both 1/λ_1_ and 1/λ_2_ begin to increase, but the effect is much more pronounced for 1/λ_1_, reflecting its greater sensitivity to changes in exchange dynamics.

**Figure 6:**
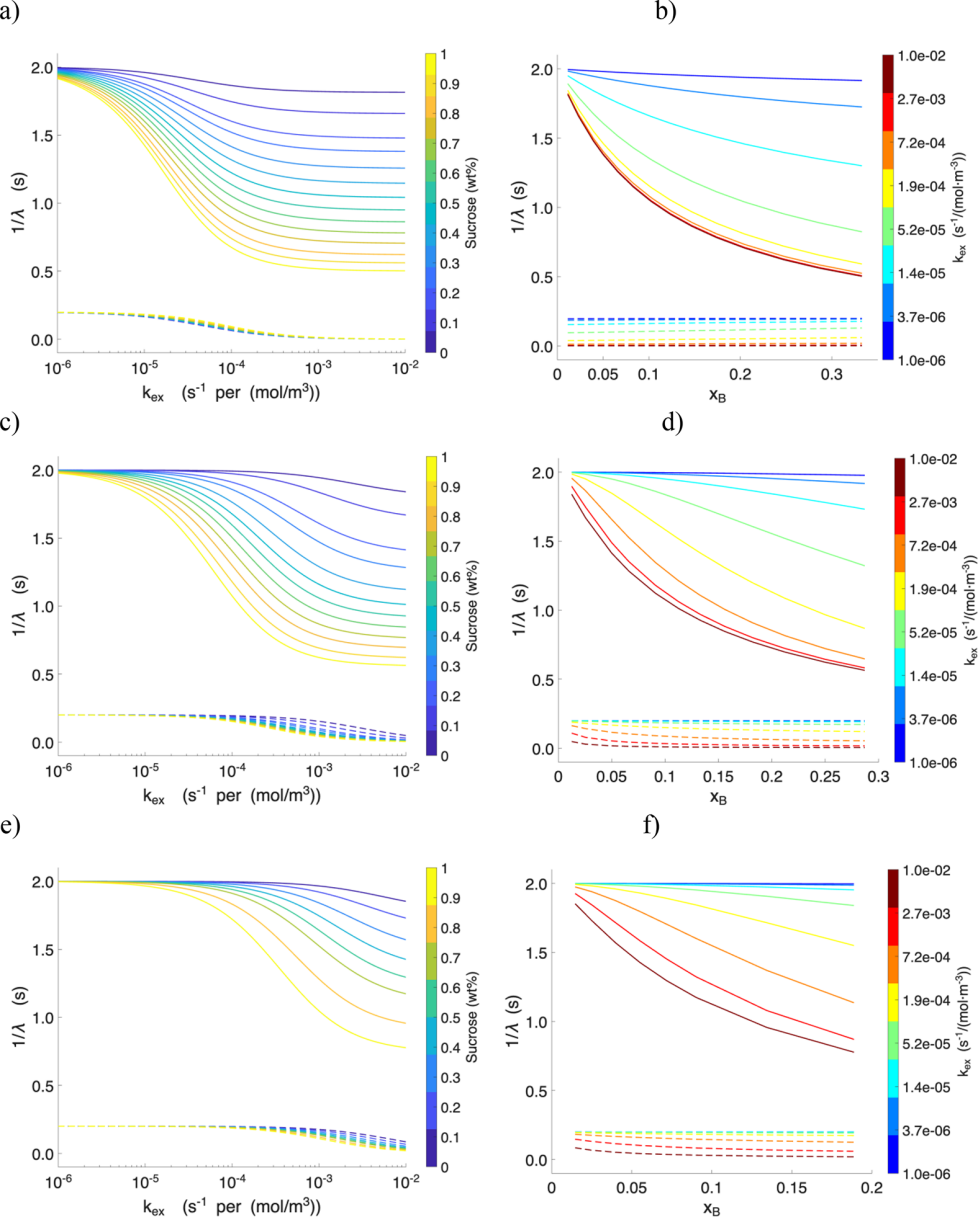
Correlation between 1/λ and the exchange rate constant (*k*_ex_) and between 1/λ and the mole fraction of the solute (*x*_*B*_): solid lines represent 1/λ_1_ and dashed lines represent 1/λ_2_. Panels (a, b) present the results for sucrose in H_2_O, panels (c, d) for sucrose in D_2_O, and panels (e, f) for freeze-dried sucrose reconstituted in D_2_O. The comparative analysis highlights the influence of solvent environment and sample preparation on the exchange dynamics and relaxation behavior.

The concentration dependence is further clarified in Figure 6b: for a fixed *k*_ex_, 1/λ_2_ remains nearly constant across the sucrose concentration range, while 1/λ_1_ decreases as the mole fraction of sucrose increases. Only at the slowest *k*_ex_ values do 1/λ_2_ exhibit a slight increase, though it remains essentially invariant with concentration. Conversely, the dependence of 1/λ_1_ on *x*_*B*_ diminishes systematically as *k*_ex_ is reduced by orders of magnitude, indicating that the relaxation response becomes less sensitive to solute concentration under slow exchange conditions.

For sucrose in D_2_O, the trends shift noticeably. Both 1/λ_1_ and 1/λ_2_ exhibit a reduced sensitivity to *k*_ex_, with the onset of the “flattening” behavior occurring nearly an order of magnitude earlier compared to sucrose in H_2_O. This shift is expected because the background proton population in D_2_O is much lower, with exchange contributions from sucrose hydroxyl groups becoming dominant. In this system, both 1/λ_1_ and 1/λ_2_ also display a somewhat stronger dependence on solute mole fraction, suggesting that solute contributions play a proportionally greater role in defining the relaxation landscape.

The freeze-dried sucrose reconstituted in D_2_O qualitatively resembles the behavior of sucrose in D_2_O, with exchange dynamics slowed relative to H_2_O and with measurable, though modest, concentration dependence in both 1/λ_1_ and 1/λ_2_. These results demonstrate that the exchange behavior is highly on the solvent, with H_2_O dominated by the fast solvent exchange, D_2_O amplifying the relative contribution of solute hydroxyls, and partially deuterated sucrose samples displaying an attenuated but qualitatively similar response. Overall, this analysis extends beyond the Bloch–McConnell two-site exchange model by explicitly demonstrating how solvent isotope composition and sample preparation affect exchange dynamics in sucrose-water systems, offering new molecular insight into proton relaxation mechanisms that is not available from standard approaches.

Finally, we point out that in a strongly deuterated system the fast exchange assumption is not necessarily valid. Indeed, *k*_*A*_ and *k*_*B*_ are products of the exchange rate by respective concentrations (for example in moles per unit of volume). In a strongly deuterated system, protons become diluted, and both *A* ^tot^ and *B* ^tot^ become very small, decreasing *k*_*A*_ and *k*_*B*_. Hence, eqs 36–39 may not accurately work for FD-D_2_O case. This can be illustrated by the slow component behavior for this system (Figures 2a and 4a, black squares) which does not continue the growing trend observed upon partial deuteration of the system.

## Conclusions

In applications to sucrose-water systems, the low-field NMR relaxation methodology shows difficulties in the distinguishing between “bound” and “free” water characteristics. This limitation arises because the NMR relaxation signals do not provide a clear delineation between water molecules tightly associated with sucrose (“bound” water) and those that remain free or loosely associated (“free” water). The observed multiexponential relaxation behavior in these systems likely results from the relaxation contributions of distinct proton populations. Specifically, these protons can be categorized as exchangeable protons—those involved in hydrogen bonding or exchange processes with the surrounding environment—and nonexchangeable protons, which are not directly engaged in such interactions. This complex relaxation behavior suggests that the relaxation dynamics of the system are influenced by both molecular interactions and exchange processes, complicating the interpretation of the data and the precise identification of the bound and unbound states of water.

## Supplementary Material



## Notes

Additional equations, tables of relaxation times, and proton concentrations, including Appendix A and Appendix B (PDF)
