# Supplementary material for: Proton Nuclear Magnetic Resonance Relaxation in Aqueous Sugar Solutions: Can Low-Field Nuclear Magnetic Resonance Relaxation Measurements Differentiate between “Bound” and “Free” Water?
Source: ACS Phys Chem Au. 2025 Nov 11;6(1):196–206. doi: 10.1021/acsphyschemau.5c00108 (PMC12856673; doi:10.1021/acsphyschemau.5c00108)
Supplement: Supplementary file 1 [file pg5c00108_si_001.pdf]

Electronic Supporting Information for

Proton Nuclear Magnetic Resonance Relaxation on Aqueous Sugar Solutions: Can Low-Field Nuclear Magnetic Resonance Relaxation Measurements Differentiate between ‘Bound’ and ‘Free’ Water?

Vasantha Gowda[†,#], Ivan Argatov[†,#], Olle Söderman[‡], Vitaly Kocherbitov[†,#]*

[†]Department of Biomedical Science, Faculty of Health and Society, Malmö University, Malmö, SE-205 06 Sweden

[#]Biofilms – Research Center for Biointerfaces, Malmö University, SE-205 06 Malmö, Sweden

[‡]Division of Physical Chemistry, Lund University, P. O. Box 124, SE-22100 Lund, Sweden

*Corresponding author at: Faculty of Health and Society, Malmö University, SE-205 06 Malmö, Sweden. E-mail address: vitaly.kocherbitov@mau.se (V. Kocherbitov).

Bloch-McConnell equations

Here we briefly remind the Bloch and the Bloch - McConnell equations used to describe and interpret NMR relaxation data.

The Bloch equations are used to calculate the nuclear magnetization $M = (M_x, M_y, M_z)$ as a function of time when longitudinal relaxation (T_1) and transverse relaxation (T_2) times are present. These are phenomenological equations that were introduced by Felix Bloch in 1946. The Bloch-McConnell equations are an extension of the Bloch equations to consider chemical exchange between different pools of spins. For a nucleus exchanging between two distinct magnetic environments, A and B, with different chemical shift and different relaxation rates, the exchange processes can be written as:

$$A \underset{k_B}{\overset{k_A}{\rightleftharpoons}} B \quad \text{(S1)}$$

where k_A and k_B are the exchange rates out of site A and B, respectively. For the longitudinal magnetization this model corresponds to the following differential equations:

$$\frac{dM_{z,A}}{dt} = R_{1,A}\left(M_{z,A}^{\infty} - M_{z,A}\right) - k_A M_{z,A} + k_B M_{z,B} \quad \text{(S2)}$$

$$\frac{dM_{z,B}}{dt} = R_{1,B}\left(M_{z,B}^{\infty} - M_{z,B}\right) - k_B M_{z,B} + k_A M_{z,A} \quad \text{(S3)}$$

Here, $R_{1,A}$ and $R_{1,B}$ are the longitudinal relaxation rates of the spins in the two sites while $M_{z,A}^{\infty}$ and $M_{z,B}^{\infty}$ are the equilibrium magnetizations.

The general solutions of equations (S2) and (S3) are

$$M_{z,A}(t) = \Lambda_1.\ e^{\lambda_1 t} + \Lambda_2.\ e^{\lambda_2 t} + M_{z,A}^{\infty} \quad \text{(S4)}$$

$$M_{z,B}(t) = \Lambda_3.\ e^{\lambda_1 t} + \Lambda_4.\ e^{\lambda_2 t} + M_{z,B}^{\infty} \quad \text{(S5)}$$

where expressions for λ_1, λ_2, Λ_1, Λ_2, Λ_3 and Λ_4 are shown below.

Analogously, Bloch-McConnell equations are written for transverse magnetization as follows:

$$\frac{dM_{xy,A}}{dt} = -(R_{2,A} - i\omega_A)M_{xy,A} - k_A M_{xy,A} + k_B M_{xy,B} \quad \text{(S6)}$$

$$\frac{dM_{xy,B}}{dt} = -(R_{2,B} - i\omega_B)M_{xy,B} - k_B M_{xy,B} + k_A M_{xy,A} \quad \text{(S7)}$$

where $R_{2,A}$ and $R_{2,B}$ are the transverse relaxation rates of the spins 'A' and 'B', in the absence of exchange, ω_A and ω_B are the Larmor frequencies of the corresponding sites, respectively.

The general solutions of equations (S6) and (S7) are

$$M_{xy,A}(t) = \Theta_1.\ e^{\upsilon_1 t} + \Theta_2.\ e^{\upsilon_2 t} \quad \text{(S8)}$$

$$M_{xy,B}(t) = \Theta_3.\ e^{\upsilon_1 t} + \Theta_4.\ e^{\upsilon_2 t} \quad \text{(S9)}$$

Longitudinal magnetisation:

The λ_1 and λ_2 are the time constants of the two exponentials:

$$\lambda_1 = \frac{1}{2}\{-(k_{1A} + k_{1B}) + \sqrt{(k_{1A} - k_{1B})^2 + 4k_A k_B}\} \quad \text{(S10)}$$

$$\lambda_2 = \frac{1}{2}\{-(k_{1A} + k_{1B}) - \sqrt{(k_{1A} - k_{1B})^2 + 4k_A k_B}\} \quad \text{(S11)}$$

where

$$k_{1A} = R_{1,A} + k_A \quad \text{(S12)}$$

$$k_{1B} = R_{1,B} + k_A \quad \text{(S13)}$$

and

$$\Lambda_1 = \{(\lambda_2 + k_{1A})(M_{z,A}^{\infty} - M_{z,A}^{0}) - k_B(M_{z,B}^{\infty} - M_{z,B}^{0})\}/(\lambda_1 - \lambda_2) \quad \text{(S14)}$$

$$\Lambda_2 = \{-(\lambda_1 + k_{1A})(M_{z,A}^{\infty} - M_{z,A}^{0}) + k_B(M_{z,B}^{\infty} - M_{z,B}^{0})\}/(\lambda_1 - \lambda_2) \quad \text{(S15)}$$

$$\Lambda_3 = \{-k_A(M_{z,A}^{\infty} - M_{z,A}^{0}) - (\lambda_1 + k_{1A})(M_{z,B}^{\infty} - M_{z,B}^{0})\}/(\lambda_1 - \lambda_2) \quad \text{(S16)}$$

$$\Lambda_4 = \{k_A(M_{z,A}^{\infty} - M_{z,A}^{0}) + (\lambda_2 + k_{1A})(M_{z,B}^{\infty} - M_{z,B}^{0})\}/(\lambda_1 - \lambda_2) \quad \text{(S17)}$$

Here, $M_{z,A}^{0}$ and $M_{z,B}^{0}$ are the initial magnetizations out of sites *A* and *B*, respectively.

By summing equations (4) and (5), we obtain

$$M_z(t) = M_z^{\infty} - a_1.\ e^{\lambda_1 t} - a_2.\ e^{\lambda_2 t} \quad \text{(S18)}$$

where, in view of (S5)–(S8), we have

$$-a_1 = \Lambda_1 + \Lambda_3$$
$$= \frac{(\lambda_2 + R_{1,A})(M_{z,A}^{\infty} - M_{z,A}^{0}) - (\lambda_1 + R_{1,A} + k_A + k_B)(M_{z,B}^{\infty} - M_{z,B}^{0})}{\lambda_1 - \lambda_2} \quad \text{(S19)}$$

$$-a_2 = \Lambda_2 + \Lambda_4$$
$$= \frac{-(\lambda_1 + R_{1,A})(M_{z,A}^{\infty} - M_{z,A}^{0}) + (\lambda_2 + R_{1,A} + k_A + k_B)(M_{z,B}^{\infty} - M_{z,B}^{0})}{\lambda_1 - \lambda_2} \quad \text{(S20)}$$

Transverse magnetisation:

$$\vartheta_1 = \frac{1}{2}\{-(k_{2A} + k_{2B}) + \sqrt{(k_{2A} - k_{2B})^2 + 4k_A k_B}\} \quad \text{(S21)}$$

$$\vartheta_2 = \frac{1}{2}\{-(k_{2A} + k_{2B}) - \sqrt{(k_{2A} - k_{2B})^2 + 4k_A k_B}\} \quad \text{(S22)}$$

Where

$$k_{2A} = R_{2,A} - i\omega_A + k_A \quad \text{(S23)}$$

$$k_{2B} = R_{2,B} - i\omega_B + k_B \quad \text{(S24)}$$

and

$$\Theta_1 = \{-(\vartheta_2 + k_{2A})M_{xy,A}^{0} + k_B M_{xy,B}^{0}\}/(\vartheta_1 - \vartheta_2) \quad \text{(S25)}$$

$$\Theta_2 = \{(\vartheta_2 + k_{2A})M_{xy,A}^{0} - k_B M_{xy,B}^{0}\}/(\vartheta_1 - \vartheta_2) \quad \text{(S26)}$$

$$\Theta_3 = \{k_A M^0_{xy,A} + (\vartheta_1 + k_{2A}) M^0_{xy,B}\}/(\vartheta_1 - \vartheta_2) \quad \text{(S27)}$$

$$\Theta_4 = \{-k_A M^0_{xy,A} - (\vartheta_1 - k_{2A}) M^0_{xy,B}\}/(\vartheta_1 - \vartheta_2) \quad \text{(S28)}$$

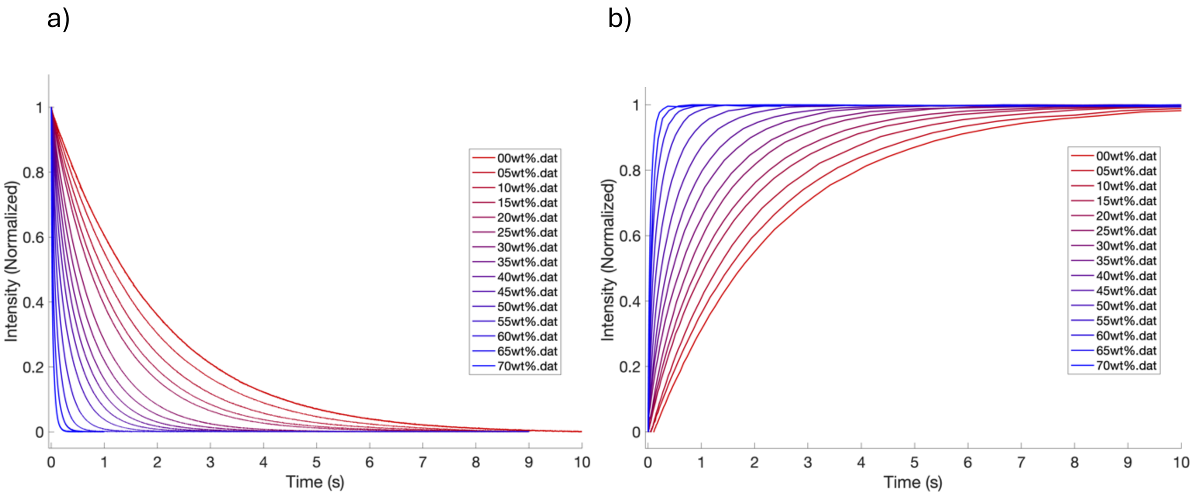

Figure S1. a) Saturation recovery [1]H T_1 a) and b) CPMG T_2 relaxation decay curves for sucrose-water systems measured at 25°C.

Table S1. [1]H T_1 relaxation times for solutions of sucrose in Milli-Q H_2O, D_2O, and freeze-dried sucrose-D_2O reconstituted in D_2O.

Milli-Q H_2O[a]					D_2O[a]					FD-D_2O[a]				
Sucrose (wt %)	T_{1f} (ms)	T_{1s} (ms)	a^n_{1f}	a^n_{1s}	Sucrose (wt %)	T_{1f} (ms)	T_{1s} (ms)	a^n_{1f}	a^n_{1s}	Sucrose (wt %)	T_{1f} (ms)	T_{1s} (ms)	a^n_{1f}	a^n_{1s}
0.00	NA	2360	0	1	NA	NA	NA	NA	NA	NA	NA	NA	NA	NA
5.02	NA	2108	0	1	5.10	481	5750	0.56	0.44	6.05	519	5247	0.87	0.13
10.14	800	1929	0.07	0.93	10.03	431	4560	0.58	0.42	10.05	426	3941	0.87	0.13
14.95	548	1718	0.08	0.92	NA	NA	NA	NA	NA	15.24	324	1941	0.85	0.15
20.02	365	1480	0.08	0.92	18.32	323	3337	0.57	0.43	20.23	266	1636	0.85	0.15
25.09	297	1244	0.10	0.90	23.17	260	2461	0.59	0.41	25.16	213	1183	0.85	0.15
30.07	220	1104	0.13	0.87	29.94	182	1892	0.59	0.41	30.13	172	960	0.85	0.15
35.17	173	913	0.17	0.83	35.16	141	1522	0.61	0.39	40.25	102	493	0.84	0.16
40.04	145	748	0.22	0.78	39.53	106	1045	0.61	0.39	50.27	21.7	50.9	0.79	0.21
45.03	104	568	0.25	0.75	44.11	76.1	693	0.61	0.39	NA	NA	NA	NA	NA
50.03	69.2	397	0.29	0.71	48.78	54.4	454	0.61	0.39	NA	NA	NA	NA	NA
55.02	49.7	255	0.32	0.68	53.52	37.3	273	0.60	0.40	NA	NA	NA	NA	NA
60.94	35.9	163	0.37	0.63	58.77	26.4	153	0.55	0.45	NA	NA	NA	NA	NA
65.58	25.6	95.6	0.39	0.61	63.24	19.9	89.0	0.45	0.55	NA	NA	NA	NA	NA
70.33	24.4	64.3	0.42	0.58	NA	NA	NA	NA	NA	NA	NA	NA	NA	NA

[a] Experiments are performed in triplicate, and the average values are reported with error bars shown in Figure 2 of the main article.

Table S2. [1]H CPMG T_2 relaxation times for solutions of sucrose in Milli-Q H_2O, D_2O (99.8%), and freeze-dried sucrose-D_2O reconstituted in D_2O (99.8%).

MilliQ H_2O[a]					D_2O (99.8%)[a]					FD D_2O (99.8%)[a]				
Sucrose (wt %)	T_2f (ms)	T_2s (ms)	$a_1^n f$	$a_1^n s$	Sucrose (wt %)	T_2f (ms)	T_2s (ms)	$a_1^n f$	$a_1^n s$	Sucrose (wt %)	T_2f (ms)	T_2s (ms)	$a_1^n f$	$a_1^n s$
0.00	NA	2005	0	1	NA	NA	NA	NA	NA	4.77	433	2384	0.83	0.17
5.02	NA	1614	0	1	5.10	427	3137	0.56	0.44	6.05	407	1836	0.83	0.17
10.14	NA	1308	0	1	10.03	350	1959	0.63	0.37	10.00	362	1535	0.85	0.15
14.95	387	1134	0.01	0.99	NA	NA	NA	NA	NA	15.24	307	1265	0.83	0.17
20.02	335	1050	0.03	0.97	18.32	279	1586	0.58	0.42	20.23	254	946	0.83	0.17
25.09	274	945	0.07	0.93	23.17	235	1270	0.58	0.42	25.16	212	803	0.80	0.20
30.07	198	737	0.09	0.91	29.94	182	982	0.60	0.40	30.13	174	663	0.84	0.16
35.17	161	681	0.14	0.86	35.16	133	750	0.59	0.41	40.25	104	443	0.86	0.14
40.04	128	562	0.18	0.82	39.53	102	600	0.59	0.41	50.27	50.6	208	0.85	0.15
45.03	94.4	452	0.21	0.79	44.11	74.5	471	0.60	0.40	NA	NA	NA	NA	NA
50.03	64.5	353	0.25	0.75	48.78	43.5	303	0.62	0.38	NA	NA	NA	NA	NA
55.02	44.1	232	0.29	0.71	53.52	34.4	232	0.61	0.39	NA	NA	NA	NA	NA
60.94	27.8	145	0.34	0.66	58.77	22.4	140	0.63	0.37	NA	NA	NA	NA	NA
65.58	16.8	82.5	0.38	0.62	63.24	13.4	78.7	0.64	0.36	NA	NA	NA	NA	NA
70.33	10.2	46.7	0.43	0.57	NA	NA	NA	NA	NA	NA	NA	NA	NA	NA

[a] Experiments are performed in triplicate, and the average values are reported with error bars shown in Figure 4 of the main article.

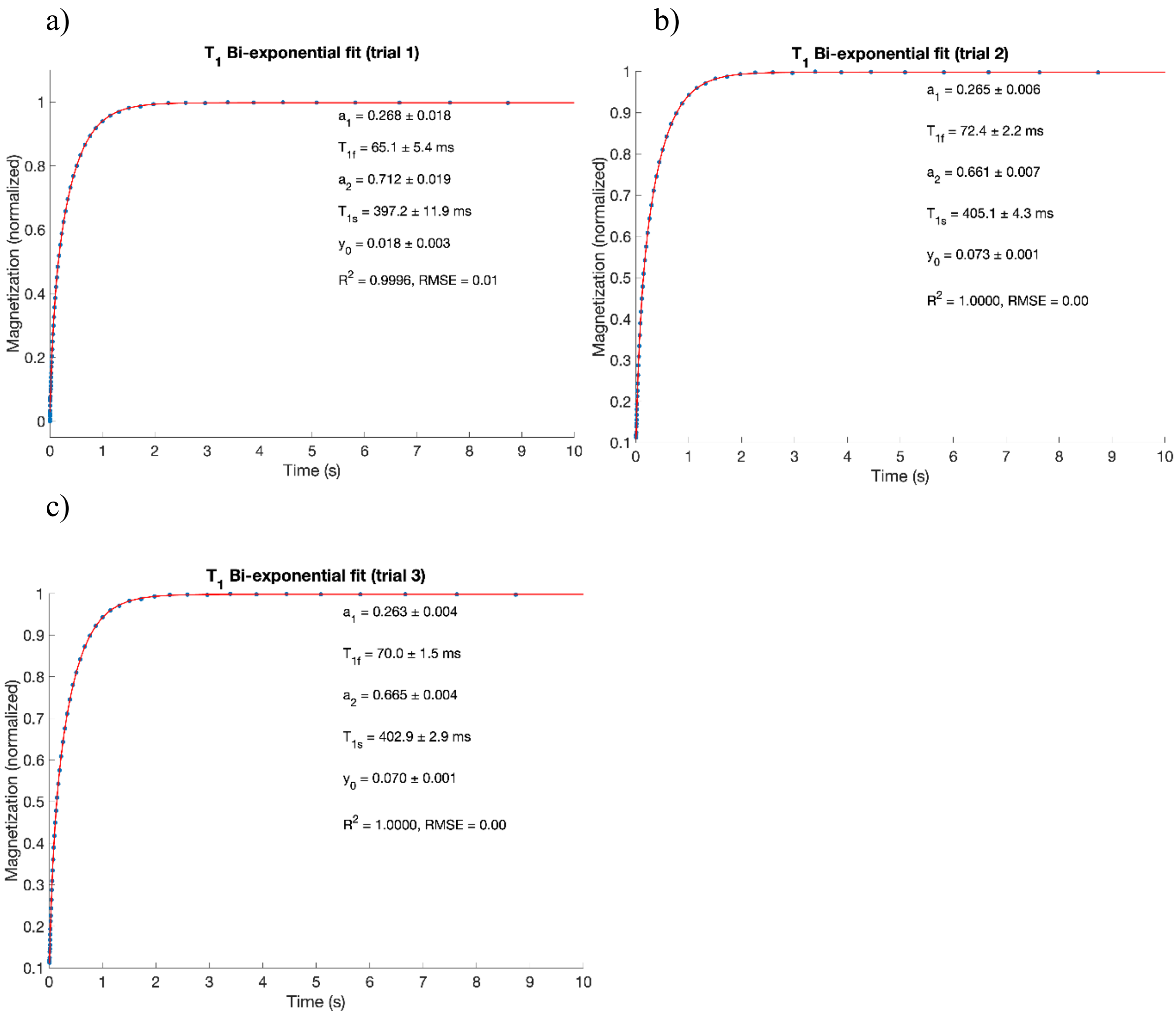

Figure S2. Bi-exponential T_1 relaxation fits for three independent trials a), b), and c) for a solution of 50 wt% sucrose in H_2O. Normalized magnetization data (blue dots) are overlaid with fitted curves (red solid lines) using a 5-parameter model. The fitted parameters show consistent trends across trials, while fitting quality is quantified by R^2 and RMSE values.

The theoretical non-exchangeable (X_P^{non}) and exchangeable (X_P^{exch}) proton contribution coefficients were calculated using the following equations:

$$X_P^{non} = \frac{\frac{m_S}{M_S}*14}{\frac{m_S}{M_S}*14+\frac{m_S}{M_S}*8+\frac{m_W}{M_W}*2} \quad \text{(S29)}$$

$$X_P^{exch} = \frac{\frac{m_S}{M_S}*8*f_S+\frac{m_W}{M_W}*2*f_H}{\frac{m_S}{M_S}*14+\frac{m_S}{M_S}*8+\frac{m_W}{M_W}*2} \quad \text{(S30)}$$

where m_s and m_w denote the masses of sucrose and water in grams, M_s and M_w represent the molecular weights of sucrose (342.3 g/mol) and water (18.015 g/mol), respectively.

In 99.8% D_2O, Eqns. S20 and S21 are expressed as:

$$X_P^{non} = \frac{\frac{m_S}{M_S}*14}{\frac{m_S}{M_S}*14+\frac{m_S}{M_S}*8*f_S+\frac{m_W}{M_W}*2*f_H} \quad \text{(S31)}$$

$$X_P^{exch} = \frac{\frac{m_S}{M_S}*8*f_S+\frac{m_W}{M_W}*2*f_H}{\frac{m_S}{M_S}*14+\frac{m_S}{M_S}*8*f_S+\frac{m_W}{M_W}*2*f_H} \quad \text{(S32)}$$

where m_w = 20.03 g/mol for D_2O.

The factors f_H and f_S specify the protonation fractions:

f_H = 1 for H_2O, f_H =0.002 for 99.8% D_2O.
f_S = 1 if fully protiated sucrose, f_S = 0.3 for freeze-dried sucrose reconstituted in D_2O, the extent of proton exchange was estimated to be 70% based on the mass difference before and after freeze-drying.

The total proton concentrations in water (A^{tot}) and sugar (B^{tot}) in the units of $\frac{mol}{m^3}$ are related to the population coefficients:

Define total solution volume as $V = \frac{m_A}{\rho_A} + \frac{m_B}{\rho_B}$

$$A^{tot} = 2\frac{m_W}{M_W*V} = 2\frac{m_W}{M_W*(V_W+V_S)} = \frac{2}{M_S\left(\frac{1}{\rho_A}+\frac{m_S}{m_W\rho_B}\right)} \quad \text{(S33)}$$

$$B^{tot} = 8\frac{m_S}{M_S*V} = 8\frac{m_S}{M_S*(V_W+V_S)} = \frac{8}{M_W\left(\frac{m_W}{m_S\rho_A}+\frac{1}{\rho_B}\right)} \quad \text{(S34)}$$

Calculations of exchangeable hydroxyl proton concentrations:

1. Sucrose-H_2O system:
   Water (A) contributes 2 exchangeable H per water molecule, therefore, the number of moles of exchangeable proton sites in sugar (in moles) = $2 * \frac{m_A}{M_A}$

   Sucrose (B) contributes 8 exchangeable H per sucrose molecule, therefore, the number of exchangeable proton sites in water (in moles) = $8 * \frac{m_B}{M_B}$

   Water proton concentration,

   $$A^{ex} = \frac{2*\frac{m_A}{M_A}}{\left(\frac{m_A}{\rho_A}+\frac{m_B}{\rho_B}\right)} = \frac{2}{M_A\left(\frac{1}{\rho_A}+\frac{m_B}{m_A*\rho_B}\right)} \text{ mol/m}^3 \quad \text{(S35)}$$

   Exchangeable sucrose proton concentration,

   $$B^{ex} = \frac{8*\frac{m_B}{M_B}}{\left(\frac{m_A}{\rho_A}+\frac{m_B}{\rho_B}\right)} = \frac{8}{M_B\left(\frac{m_A}{m_B*\rho_A}+\frac{1}{\rho_B}\right)} \text{ mol/m}^3 \quad \text{(S36)}$$

   M_B and M_A represent the molecular weights of sucrose (0.3423 kg/mol) and water (0.018015 kg/mol), respectively. ρ_B = 1587 kg/m^3, ρ_A = 997 kg/m^3

2. Sucrose-D_2O system:
   f_H : fraction of proton per hydrogen site in water (f_H = 0.002 for 99.8% D_2O)
   f_S : fraction of proton per hydrogen site in sugar (f_S = 1 for fully protiated sucrose and f_S = 0.3 for deuterated sucrose)

   Total No. of exchangeable proton sites (in moles) $= 2 * \frac{m_A}{M_A} + 8 * \frac{m_B}{M_B}$

   No. of moles of protons provided by water to the system: $= 2f_H * \frac{m_A}{M_A}$,

   No. of moles of protons provided by sugar to the system: $= 8f_s * \frac{m_B}{M_B}$,

   Total number of moles of exchangeable protons in the system:

   $$= 2 * f_H * \frac{m_A}{M_A} + 8 * f_s * \frac{m_B}{M_B}$$

   Exchangeable water proton concentration:

$$A^{ex} = \frac{\left(2*f_H*\frac{m_A}{M_A}+8*f_S*\frac{m_B}{M_B}\right)}{\left(\frac{m_A}{\rho_A}+\frac{m_B}{\rho_B}\right)} \frac{\left(2*\frac{m_A}{M_A}\right)}{\left(2*\frac{m_A}{M_A}+8*\frac{m_B}{M_B}\right)} \tag{S37}$$

Exchangeable sucrose proton concentration:

$$B^{ex} = \frac{\left(2*f_H*\frac{m_A}{M_A}+8*f_S*\frac{m_B}{M_B}\right)}{\left(\frac{m_A}{\rho_A}+\frac{m_B}{\rho_B}\right)} \frac{\left(8*\frac{m_B}{M_B}\right)}{\left(2*\frac{m_A}{M_A}+8*\frac{m_B}{M_B}\right)} \tag{S38}$$

Eqns. (S37) and (S38) reduce to eqn. (S35) and (S36), when $f_H = 1$ and $f_s = 1$, respectively.

Table S3. Theoretical proton fractions in sucrose-water, sucrose-D_2O, and freeze-dried sucrose - D_2O solutions.

Sucrose in Milli-Q water									
**Wt. Suc (mg)**	**Wt. H2O (mg)**	**Wt% Suc**	n_P^{non}	n_P^{exch} suc	n_P^{exch} water	n_P^{exch} suc+water	n_P^{total}	**X_non**	**X_exch**
54.9	1006.0	5.17	0.0022	0.0013	0.1117	0.1130	0.1152	0.02	0.98
111.8	1004.5	10.02	0.0046	0.0026	0.1115	0.1141	0.1187	0.04	0.96
113.5	1006.0	10.14	0.0046	0.0027	0.1117	0.1143	0.1190	0.04	0.96
194.3	1000.0	16.27	0.0079	0.0045	0.1110	0.1156	0.1235	0.06	0.94
250.3	1000.0	20.02	0.0102	0.0058	0.1110	0.1169	0.1271	0.08	0.92
335.0	1000.0	25.09	0.0137	0.0078	0.1110	0.1188	0.1325	0.10	0.90
430.0	1000.0	30.07	0.0176	0.0100	0.1110	0.1211	0.1387	0.13	0.87
542.5	1000.0	35.17	0.0222	0.0127	0.1110	0.1237	0.1459	0.15	0.85
667.7	1000.0	40.04	0.0273	0.0156	0.1110	0.1266	0.1539	0.18	0.82
819.2	1000.0	45.03	0.0335	0.0191	0.1110	0.1302	0.1637	0.20	0.80
1001.2	1000.0	50.03	0.0409	0.0234	0.1110	0.1344	0.1754	0.23	0.77
1223.3	1000.0	55.02	0.0500	0.0286	0.1110	0.1396	0.1896	0.26	0.74
1560.0	1000.0	60.94	0.0638	0.0365	0.1110	0.1475	0.2113	0.30	0.70
1905.0	1000.0	65.58	0.0779	0.0445	0.1110	0.1555	0.2335	0.33	0.67
2370.0	1000.0	70.33	0.0969	0.0554	0.1110	0.1664	0.2633	0.37	0.63
Sucrose in D_2O (99.8%)									
**Wt. Suc (mg)**	**Wt. D2O (mg)**	**Wt% Suc**	n_P^{non}	n_P^{exch} suc	n_P^{exch} D_2O	n_P^{exch} suc+D_2O	n_P^{total}	**X_non**	**X_exch**
26.8	1106.6	2.36	0.0011	0.0006	0.0001	0.0007	0.0018	0.60	0.40
60.0	1115.7	5.10	0.0025	0.0014	0.0001	0.0015	0.0040	0.62	0.38
124.0	1115.7	10.00	0.0051	0.0029	0.0001	0.0030	0.0081	0.63	0.37
196.5	1115.7	14.97	0.0080	0.0046	0.0001	0.0047	0.0127	0.63	0.37
250.3	1115.7	18.32	0.0102	0.0058	0.0001	0.0060	0.0162	0.63	0.37
336.4	1115.7	23.17	0.0138	0.0079	0.0001	0.0080	0.0217	0.63	0.37
477.0	1116	29.94	0.0195	0.0111	0.0001	0.0113	0.0308	0.63	0.37
605.2	1116	35.16	0.0248	0.0141	0.0001	0.0143	0.0390	0.63	0.37
729.4	1116	39.53	0.0298	0.0170	0.0001	0.0172	0.0470	0.63	0.37
880.9	1116	44.11	0.0360	0.0206	0.0001	0.0207	0.0567	0.64	0.36
1062.7	1116	48.78	0.0435	0.0248	0.0001	0.0249	0.0684	0.64	0.36
1284.8	1116	53.52	0.0525	0.0300	0.0001	0.0301	0.0827	0.64	0.36
1590.8	1116	58.77	0.0651	0.0372	0.0001	0.0373	0.1024	0.64	0.36
1919.8	1116	63.24	0.0785	0.0449	0.0001	0.0450	0.1235	0.64	0.36
Freeze dried sucrose in D_2O (99.8%)									
**Wt. Suc (mg)**	**Wt. D2O (mg)**	**Wt% Suc**	n_P^{non}	n_P^{exch} FD-suc	n_P^{exch} D_2O	n_P^{exch} FD-suc+D_2O	n_P^{total}	**X_non**	**X_exch**
64.3	998.1	6.05	0.0026	0.0004	0.0001	0.0005	0.0031	0.83	0.17
123.4	1104.2	10.05	0.0050	0.0009	0.0001	0.0010	0.0059	0.84	0.16
182.6	1015.6	15.24	0.0073	0.0013	0.0001	0.0014	0.0087	0.84	0.16
257.6	1015.6	20.23	0.0104	0.0018	0.0001	0.0019	0.0122	0.85	0.15
341.4	1015.6	25.16	0.0137	0.0024	0.0001	0.0025	0.0162	0.85	0.15
438.0	1015.6	30.13	0.0176	0.0030	0.0001	0.0031	0.0207	0.85	0.15
684.2	1015.6	40.25	0.0275	0.0047	0.0001	0.0048	0.0324	0.85	0.15
1026.6	1015.6	50.27	0.0413	0.0071	0.0001	0.0072	0.0485	0.85	0.15

Table S4. Exchangeable proton concentration in sucrose-water system. T_{1s} $(1/\lambda_1)$, T_{1f} $(1/\lambda_2)$, R_{1A}=0.424 s^{-1} (T_{1s}=2.36 s), R_{1B}=7.3 s^{-1}(average) (T_{1f}=0.14 s)

Sucrose (wt%)	A^{ex} (mol/m^3)	B^{ex} (mol/m^3)	x*A*	x*B*	k_B (s^{-1})	k_A (s^{-1})	T_{1s} (s) Exact	T_{1f} (s) Exact	T_{1s} (s) FE	T_{1f} (s) FE
5.2	1.07E+05	1.23E+03	0.989	0.011	2140.3	24.6	2.10	4.61E-04	2.10	4.61E-04
10.0	1.03E+05	2.42E+03	0.977	0.023	2069.0	48.5	1.89	4.71E-04	1.89	4.71E-04
16.3	9.86E+04	4.03E+03	0.961	0.039	1972.9	80.7	1.66	4.86E-04	1.66	4.86E-04
20.0	9.56E+04	5.04E+03	0.950	0.050	1912.9	100.8	1.53	4.95E-04	1.53	4.95E-04
25.1	9.14E+04	6.45E+03	0.934	0.066	1828.8	129.0	1.38	5.10E-04	1.38	5.10E-04
30.1	8.71E+04	7.89E+03	0.917	0.083	1742.9	157.8	1.21	5.25E-04	1.21	5.25E-04
35.2	8.26E+04	9.43E+03	0.898	0.103	1651.0	188.6	1.02	5.42E-04	1.02	5.42E-04
40.0	7.80E+04	1.10E+04	0.877	0.123	1559.5	219.2	0.78	5.60E-04	0.78	5.60E-04
45.0	7.31E+04	1.26E+04	0.853	0.147	1461.5	252.1	0.53	5.81E-04	0.53	5.81E-04
50.0	6.79E+04	1.43E+04	0.826	0.174	1358.9	286.4	0.35	6.03E-04	0.34	6.03E-04
55.0	6.26E+04	1.61E+04	0.795	0.205	1251.7	322.4	0.22	6.29E-04	0.22	6.29E-04
60.9	5.59E+04	1.84E+04	0.753	0.247	1118.0	367.2	0.14	6.63E-04	0.13	6.64E-04
65.6	5.04E+04	2.02E+04	0.714	0.286	1007.7	404.1	0.10	6.96E-04	0.09	6.96E-04
70.3	4.45E+04	2.22E+04	0.667	0.333	889.4	443.8	0.07	7.35E-04	0.07	7.35E-04

Table S5. Exchangeable proton concentration in sucrose-D_2O system. T_{1s} $(1/\lambda_1)$, T_{1f} $(1/\lambda_2)$ R_{1A}=0.143 s^{-1}(T_{1s} = 7s) , R_{1B} = 8.263 s^{-1} (average)(T_{1f} = 0.12s)

Sucrose (wt%)	A^{ex} (mol/m^3)	B^{ex} (mol/m^3)	x*A*	x*B*	k_B (s^{-1})	k_A (s^{-1})	T_{1s} (s) Exact	T_{1f} (s) Exact	T_{1s} (s) FE	T_{1f} (s) FE
5.1	1.53E+03	1.93E+01	0.988	0.012	30.62	0.39	6.04	3.03E-02	5.99	3.03E-02
10.0	2.79E+03	7.27E+01	0.975	0.025	55.88	1.45	5.10	1.68E-02	5.05	1.68E-02
18.3	4.94E+03	2.59E+02	0.950	0.050	98.77	5.19	3.49	9.35E-03	3.45	9.35E-03
23.2	6.18E+03	4.36E+02	0.934	0.066	123.54	8.72	2.63	7.36E-03	2.58	7.36E-03
29.9	7.88E+03	7.88E+02	0.909	0.091	157.65	15.77	1.62	5.60E-03	1.59	5.60E-03
35.2	9.16E+03	1.16E+03	0.887	0.113	183.15	23.25	1.11	4.70E-03	1.08	4.70E-03
39.5	1.02E+04	1.56E+03	0.867	0.133	203.73	31.16	0.75	4.11E-03	0.73	4.11E-03
44.1	1.12E+04	2.07E+03	0.844	0.156	224.35	41.45	0.48	3.61E-03	0.46	3.61E-03
48.8	1.22E+04	2.72E+03	0.818	0.182	243.89	54.35	0.30	3.19E-03	0.29	3.19E-03
53.5	1.31E+04	3.53E+03	0.788	0.212	261.80	70.54	0.18	2.83E-03	0.17	2.83E-03
58.8	1.39E+04	4.65E+03	0.750	0.250	278.61	92.95	0.11	2.50E-03	0.10	2.50E-03
63.2	1.45E+04	5.83E+03	0.713	0.287	289.55	116.57	0.08	2.26E-03	0.07	2.26E-03

Table S6. Exchangeable proton concentration in FD-sucrose-D_2O system. T_{1s} $(1/\lambda_1)$, T_{1f} $(1/\lambda_2)$ R_{1A}=0.143 s^{-1}(T_{1s} = 7s) , R_{1B} = 4.227 s^{-1} (average) (T_{1f} = 0.237s)

Sucrose (wt%)	A^{ex} (mol/m^3)	B^{ex} (mol/m^3)	x*A*	x*B*	k_B (s^{-1})	k_A (s^{-1})	T_{1s} (s) Exact	T_{1f} (s) Exact	T_{1s} (s) FE	T_{1f} (s) FE
6.1	678.1	10.2	0.985	0.015	13.6	0.2	6.0	6.38E-02	5.9	6.38E-02
10.1	981.58	25.7	0.975	0.025	19.6	0.5	5.2	4.46E-02	5.0	4.46E-02
15.2	1375.1	57.9	0.960	0.040	27.5	1.2	4.0	3.16E-02	3.8	3.16E-02
20.2	1752.3	104.0	0.944	0.056	35.0	2.1	3.0	2.46E-02	2.9	2.46E-02
25.2	2120.8	166.9	0.927	0.073	42.4	3.3	2.2	1.99E-02	2.1	2.00E-02
30.1	2486.6	251.0	0.908	0.092	49.7	5.0	1.6	1.66E-02	1.5	1.67E-02
40.3	3196.8	504.0	0.864	0.136	63.9	10.1	0.8	1.21E-02	0.7	1.21E-02
50.3	3819.8	903.7	0.809	0.191	76.4	18.1	0.2	7.44E-03	0.1	7.59E-03

Appendix A. Leading-order asymptotic analysis. Longitudinal case

Let us recall that $k_A = k_{\mathrm{ex}} \cdot A^{\mathrm{tot}}$ and $k_B = k_{\mathrm{ex}} \cdot B^{tot}$, where A^{tot} and B^{tot} are the total concentrations of protons from water (A^{tot}) and sucrose (B^{tot}).

We start by observing that the coefficients of the differential (14) and (15) have the same dimension of the inverse time. In certain situations, the rate coefficients $R_{1,A}$ and $R_{1,B}$ can be regarded to be much less than the exchange rate coefficient k_{ex}, that is $R_{1,A} \ll k_{\mathrm{ex}}$ and $R_{1,B} \ll k_{\mathrm{ex}}$. In such a case, formulas (19) and (20) can be asymptotically simplified as follows:

$$\lambda_1 \cong \frac{k_A R_{1A} + k_B R_{1B}}{k_A + k_B} \quad \text{(A1)}$$

$$\lambda_2 \cong k_A + k_B + \frac{k_B R_{1A} + k_A R_{1B}}{k_A + k_B} \quad \text{(A2)}$$

In other words, we obtain that $\lambda_1 \ll \lambda_2$.

Further, by summing Eqs. (16) and (17), we can write

$$M_{z,A}(t) + M_{z,B}(t) = -C_1\, e^{-\lambda_1 t} - C_2\, e^{-\lambda_2 t} + M_{z,A}^{\infty} + M_{z,B}^{\infty} \quad \text{(A3)}$$

where the coefficients C_1 and C_2 can be expressed in terms of Λ_1 and Λ_2 (see Eqs. (21) and (22)). However, in the special case under consideration, in view of the asymptotic formulas (A1) and (A2), it can be shown that up to the second-order small terms the following estimates take place:

$$\frac{C_1}{M_{z,A}^{\infty} + M_{z,B}^{\infty}} \cong 1 \quad \text{(A4)}$$

$$\frac{C_2}{M_{z,A}^{\infty} + M_{z,B}^{\infty}} \cong 0 \quad \text{(A5)}$$

Appendix B. Leading-order asymptotic analysis. Transverse case

To simplify our analysis, we put $\omega_A = \omega_B = \omega$. Then, under the assumtion that $R_{1,A} \ll k_{\mathrm{ex}}$ and $R_{1,B} \ll k_{\mathrm{ex}}$, from Eqs. (30) and (31), it follows that

$$\vartheta_1 \cong \frac{k_A R_{2A} + k_B R_{2B}}{k_A + k_B} - i\omega \quad \text{(B1)}$$

$$\vartheta_2 \cong k_A + k_B + \frac{k_B R_{2A} + k_A R_{2B}}{k_A + k_B} - i\omega \quad \text{(B2)}$$

Further, by summing Eqs. (28) and (29), we can write

$$M_{xy,A}(t) + M_{xy,B}(t) = D_1\, e^{-\vartheta_1 t} + D_2\, e^{-\vartheta_2 t} \quad \text{(B3)}$$

where the coefficients D_1 and D_2 can be expressed in terms of Θ_1 and Θ_2 (see Eqs. (34) and (35)). However, in the special case under consideration, in view of the asymptotic formulas (B1) and (B2), it can be shown that up to the second-order small terms the following estimates take place:

$$\frac{D_1}{M_{xy,A}^{0} + M_{xy,B}^{0}} \cong 1 \quad \text{(B4)}$$

$$\frac{D_2}{M_{xy,A}^{0} + M_{xy,B}^{0}} \cong 0 \quad \text{(B5)}$$
